# Politik auf der Straße? Zum Forschungsstand zu sozialen Bewegungen, Protest und Zivilgesellschaft

**DOI:** 10.1007/s12286-022-00542-8

**Published:** 2022-11-30

**Authors:** Manès Weisskircher

**Affiliations:** 1 Department of Sociology and Human Geography, Universität Oslo, Oslo, Norwegen; 2 Center for Research on Extremism (C-REX), Universität Oslo, Oslo, Norwegen

## Abstract

Soziale Bewegungen prägen zeitgenössische Gesellschaften. Dieser Beitrag diskutiert die wesentlichen Erkenntnisse der internationalen Bewegungs- und Protestforschung und ordnet den Forschungsstand ein. Das Ziel ist es, die Aufmerksamkeit sozialwissenschaftlicher Forschung verstärkt auf Politik „auf der Straße“ zu lenken. Dabei fokussiert der Artikel auf vier zentrale Fragestellungen: die definitorische Annäherung an das Phänomenon (was sind soziale Bewegungen?), ihre Formierung (wann und warum entstehen soziale Bewegungen?), ihre Aktionsformen (wie agieren soziale Bewegungen?) sowie ihre Auswirkungen (welchen Einfluss haben soziale Bewegungen?). Abschließend plädiert der Beitrag für eine enge Verknüpfung von Bewegungs- und Protestforschung mit der Parteienforschung und der politischen Soziologie. Protest ist nicht bloß *l’art pour l’art*: Ohne eine Bezugnahme auf Parteipolitik und Gesellschaftsanalyse bleibt das Verständnis von sozialen Bewegungen begrenzt. Andererseits würde die Analyse von (Partei‑)Politik und gesellschaftlichen Makro-Entwicklungen von einer verstärkten Berücksichtigung sozialer Bewegungen profitieren.

## Einleitung

Soziale Bewegungen prägen zeitgenössische Gesellschaften in (beinahe) jedem politischen Kontext. Auch für das generelle Verständnis der deutschen (Partei‑)Politik ist ein Blick auf die Protestarena essenziell, beispielsweise um die Salienz neuer Themen sowie die Entwicklung alter und neuer gesellschaftlicher Konfliktlinien zu analysieren und die Entstehung neuer Parteien zu verstehen (Hutter und Weisskircher 2022). Fridays for Future, PEGIDA, #unteilbar und Querdenken sind nur einige soziale Bewegungsorganisationen, die in der jüngsten Vergangenheit den Parteienwettbewerb und die gesellschaftliche Debatte mitbestimmten. Doch auch das Erbe vergangener Straßenmobilisierung wirft weiterhin lange Schatten auf heutige Verhältnisse, wie die „Friedliche Revolution“ in der DDR, feministische Proteste, die „Achtundsechziger“, die ArbeiterInnenbewegung oder gar die Französische Revolution. Laut mancher ForscherInnen leben wir mittlerweile in „Bewegungsgesellschaften“ (z. B. Rucht und Neidhardt 2002).

Die akademische Bewegungs- und Protestforschung bildet in vielen Ländern Europas und den Amerikas eine lebhafte „scientific community“. Sie befindet sich zumeist an der Schnittstelle von Politikwissenschaft und Soziologie mit eigenen Lehrstühlen und Forschungszentren. Die Theoriedebatten der vergangenen Jahrzehnte sind vor allem, aber nicht nur in den Vereinigten Staaten geprägt worden. In Deutschland ist die Institutionalisierung des Forschungsfeld noch weniger stark ausgeprägt, was auch mit der Staatszentriertheit der Politikwissenschaft und der Marginalisierung der politischen Linken an den Universitäten in den Nachkriegsjahrzehnten zusammenhängt (Haunss 2016). In jüngster Vergangenheit ist diesbezüglich jedoch einiges in Bewegung: Die Forschung zur deutschen Protestlandschaft an politikwissenschaftlichen und soziologischen Instituten wächst und nimmt dabei meist auf international diskutierte Theorien Bezug und geht dabei methodologisch qualitativ und quantitativ vor (Roth und Rucht 2008; Rucht 2000; siehe auch Daphi et al. 2017; Hutter und Weisskircher 2022; Opp 2009; Walter 2013).

Dieser Beitrag diskutiert die wesentlichen Erkenntnisse der internationalen Bewegungs- und Protestforschung und ordnet den Forschungsstand ein. Das Ziel ist es, die Aufmerksamkeit sozialwissenschaftlicher Forschung verstärkt auf Politik „auf der Straße“ zu lenken. Dabei fokussiert der Artikel auf vier zentrale Fragestellungen: die definitorische Annäherung an das Phänomenon (was sind soziale Bewegungen?), ihre Formierung (wann und warum entstehen soziale Bewegungen?), ihre Aktionsformen (wie agieren soziale Bewegungen?) sowie ihre Auswirkungen (welchen Einfluss haben soziale Bewegungen?).

Abschließend plädiert der Beitrag für eine enge Verknüpfung von Bewegungs- und Protestforschung mit Parteienforschung und Soziologie. Protest ist nicht bloß *l’art pour l’art*: Ohne eine Bezugnahme auf Parteipolitik und Gesellschaftsanalyse bleibt das Verständnis von sozialen Bewegungen begrenzt. Andererseits würde die Analyse von (Partei‑)Politik und gesellschaftlichen Makro-Entwicklungen von einer verstärkten Berücksichtigung von sozialen Bewegungen profitieren.

## Was ist eine soziale Bewegung?

Die empirische Literatur zu politischem Aktivismus jenseits der Wahlarena stützt sich zumeist auf den Begriff der sozialen Bewegung, deutlich seltener auf den der Zivilgesellschaft. Die Forschung bietet dabei eine Reihe an Definitionen an (siehe Tab. 1). Soziale Bewegungen können u. a. als *change agent*, kollektiver Akteur, Netzwerk, öffentliche Meinung oder als rhetorische Selbstermächtigung verstanden werden. Die am häufigsten zitierte Definition ist der Netzwerk-Ansatz von Diani (1992, S. 13): Soziale Bewegungen werden dabei als „*network of informal interactions between a plurality of individuals, groups and/or organizations, engaged in a political or cultural conflict, on the basis of a shared collective identity*“ verstanden. Die Umweltbewegung kann hierfür zur Anschauung dienen: Sie besteht aus einem breiten Netzwerk an Individuen wie Greta Thunberg, Jennifer Morgan und Luisa Neubauer und vielen anderen, weniger bekannten AktivistInnen und UnterstützerInnen, informellen Gruppen auf lokaler Ebene und formalen Organisationen wie Greenpeace, Sea Shepherd und dem WWF (beide werden unabhängig vom Organisationsgrad oft als *social movement organizations [SMOs]* tituliert), die in einem politischen Konflikt engagiert sind und sich einander zugehörig fühlen.

**Tab. 1:** Auswahl an Definitionen sozialer Bewegungen in der wissenschaftlichen Literatur. (Adaptiert auf Basis von Opp 2009, S. 35)

“A social movement is a purposive and collective attempt of a number of people to change individuals or societal institutions and structures” (Zald und Ash 1966, S. 329).
A “social movement” is “a set of opinions and beliefs in a population which represents preferences for changing some elements of the social structure and/or reward distribution of a society” (McCarthy und Zald 1977, S. 1217–1218).
“A social movement is a network of informal interactions between a plurality of individuals, groups and/or organizations, engaged in a political or cultural conflict, on the basis of a shared collective identity” (Diani 1992, S. 13).
“social movements can be thought of as collectivities acting with some degree of organization and continuity outside of institutional or organizational channels for the purpose of challenging or defending extant authority, whether it is institutionally or culturally based, in the group, organization, society, culture, or world order of which they are a part” (Snow et al. 2004, S. 11).
Social movements are “collective challenges, based on common purposes and social solidarities, in sustained interaction with elites, opponents, and authorities” (Tarrow 1994, S. 9).
“social movement[s] emerged from an innovative, consequential synthesis of three elements: 1. a sustained, organized public effort making collective claims on target authorities (let us call it a *campaign*); 2. employment of combinations from among the following forms of political action: creation of special-purpose associations and coalitions, public meetings, solemn processions, vigils, rallies, demonstrations, petition drives, statements to and in public media, and pamphleteering (call the variable ensemble of performances the *social movement repertoire*); and participants’ concerted public representations of WUNC: worthiness, unity, numbers, and commitment on the part of themselves and/or their constituencies (call them *WUNC displays*)” (Tilly 2004, S. 3–4).
“Social movements have traditionally been defined as organized efforts to bring about social change” (Jenkins und Form 2005, S. 331).
“The label ‘social movement’ is usually a rhetorical claim by a network of individuals and organizations that wish to project more unity of purpose and strategy than they actually share, or greater strength and national (or even international) breadth” (Jasper et al. 2022, S. 21).

Wenngleich Dianis Netzwerk-Definition eine hilfreiche Annäherung an den Untersuchungsgegenstand bietet, bleiben einige Fragen offen, die die Komplexität sozialer Bewegungen verdeutlichen: (1) Wie sind verschiedene Bewegungen sinnvoll voneinander abzugrenzen? Sind die Klimaschutzbewegung, die Anti-AKW-Bewegung oder gar die Tierschutzbewegung Teil der Umweltbewegung oder präziser als separate Bewegungen zu klassifizieren? (2) Eng mit dem vorherigen Punkt verknüpft: Inwiefern gibt es die von Diani vermuteten geteilten kollektiven Identitäten tatsächlich? Einige der AkteurInnen, die von außen als relativ homogene Bewegung erscheinen, sind tief gespalten und stehen einander skeptisch oder gar ablehnend gegenüber, gerade auch in „der“ Umweltbewegung (Saunders 2008). (3) Verfolgen soziale Bewegungsorganisationen auch Aktionsformen, die typischerweise mit Interessengruppen (z. B. Lobbying wie im Falle des Naturschutzbund Deutschland) oder gar Parteien (z. B. Wahlantritt wie im Falle der Klimaliste) in Verbindung stehen? Während Dianis Definition nicht auf Aktionsformen Bezug nimmt, macht er jedenfalls explizit, dass auch Parteien, z. B. die Grünen, Teil der „plurality of […] organizations“ (Diani 1992, S. 15) sein können, die eine soziale Bewegung ausmachen. Auch Dianis konzeptuelle Annäherung kann soziale Bewegungen definitorisch nicht vollständig fassen. Dies ist der *nature of the beast* geschuldet – soziale Bewegungen sind fluide gesellschaftliche Phänomene.^1^

Zentral bei der Frage der Konzeptualisierung ist, dass die Forschung einen primär empirischen Blick auf soziale Bewegungen und Protest wirft (Edwards 2014) – im Sinne von Tocquevilles (1835/1840/1986) Betrachtungen über die „politischen Vereinigungen“ in seinem Hauptwerk „Über die Demokratie in Amerika“. Eine empirische Perspektive zeigt, dass soziale Mobilisierung in vielen Fällen zur Demokratiequalität beitragen kann (Putnam 2000), diese aber auch zu gefährden vermag, wie es beispielsweise in der Weimarer Republik der Fall war (Berman 1997). Die Forschung verfängt sich daher nicht mehr in Debatten, ob Rechtsaußen-Protest oder gewalttätiger Protest ebenso Teil eines Verständnisses von „Zivilgesellschaft“ sein *sollen* (eine kantige Ablehnung der Vermischung normativer und empirischer Perspektiven liefert Clifford 2011), sondern betont, dass unterschiedliche protestierende kollektive Akteure mit demselben theoretischen und methodologischen Rüstzeug zu verstehen sind (della Porta und Diani 2011).

Nachdem die Realität sozialer Bewegungen zumindest in Westeuropa und den Amerikas eine „progressive“ ist – in den Vereinigten Staaten war die Erforschung der afro-amerikanischen Bürgerrechtsbewegung prägend (insbesondere McAdam 1982) –, überwiegen Forschungsarbeiten zu linken und liberalen Akteuren. In Westeuropa ist es die (breit verstandene) politische Linke, die die Protestlandschaft der letzten Jahrzehnte dominierte (Hutter 2014b; Torcal et al. 2016). Parallel zum starken Anstieg der Forschung zum überwiegend parteipolitischen Rechtspopulismus (für einen Überblick siehe Heinze 2022) ist mittlerweile auch Rechtsaußen-Aktivismus von zunehmenden Forschungsinteresse (Beau Segers 2022; Berntzen und Weisskircher 2016; Caiani et al. 2012; Castelli Gattinara und Pirro 2019; Jäckle und König 2017). Eine breite Perspektive entspricht auch der Realität in anderen Kontexten: in mittel- und osteuropäischen Ländern ist Straßenprotest nicht notwendigerweise links konnotiert (Borbáth und Gessler 2020). Auch im Bereich des Online-Aktivismus prägt, trotz Technooptimismus in den Anfangsjahren des Internet, mittlerweile der unterschiedlich stark organisierte Hass von Rechtsaußen-Akteuren die Debatte (Fielitz and Marcks 2020; Winter 2019).

Jenseits der Links-Rechts-Unterscheidung gibt es weitere relevante Klassifikationen. Als *neue soziale Bewegungen* werden in der Regel die postmaterialistischen Bewegungen der Mittelschicht seit den 1960er Jahren verstanden, die sich nicht mehr primär auf sozioökonomische Anliegen fokussieren und von der ArbeiterInnenklasse getragen werden, sondern gesellschaftlichen Liberalismus (z. B. Frauenrechte, LGBT-Rechte) oder ökologische Themen betonen und verstärkt auf Veränderungen jenseits staatlicher Politik abzielen (Melucci 1980; Offe 1985). Diese vermeintlich neuen Bewegungen haben jedoch oftmals eine lange Vorgeschichte: Pointiert diskutiert Calhoun (1993) die Relevanz der „‚neuen sozialen Bewegungen‘ im frühen neunzehnten Jahrhundert“. Beispielsweise wurde die älteste noch existierende Tierschutzorganisation der Welt – die Royal Society for the Prevention of Cruelty to Animals – bereits im Jahr 1824 gegründet. Als *Not-In-My-Backyard-Proteste (NIMBY)* gelten solche, die anlassbezogen gegen lokale Infrastrukturprojekte mobilisieren, ohne eine prinzipielle, ideologisch fundierte Ablehnung ebendieser. KritikerInnen des NIMBY-Konzepts betonen jedoch, dass lokale Protestgruppen nicht bloß als Ansammlung egoistischer WutbürgerInnen zu verstehen sind, sondern ihre Anliegen häufig gesamtgesellschaftlich begründen und sich oftmals breiter vernetzen (Gordon und Jasper 1996). Das Konzept der *Bewegungspartei* (Kitschelt 2006) hat vor allem im Kontext der Anti-Austeritäts-Protestwelle in Südeuropa Aufwind genommen (della Porta et al. 2017). *Revolutionäre Bewegungen* sind ein weiterer Spezialfall sozialer Bewegungen, deren wissenschaftliche Diskussion jedoch nur zum Teil in die Bewegungsliteratur eingebettet ist (siehe z. B. Goldstone und Ritter 2018; Goodwin 2001; Kurzman 1996).

## Wann entstehen und wie entwickeln sich soziale Bewegungen?

Die Frage nach der Entstehung sozialer Bewegungen hat die akademische Bewegungs- und Protestforschung über einen langen Zeitraum geprägt, wobei Faktoren wie Struktur, Ressourcen, Kultur, Mechanismen und Agency maßgeblich diskutiert wurden. Zu Beginn des modernen Kanons^2^ sozialwissenschaftlicher Forschung zu kollektivem Handeln stand eine wesentliche Erkenntnis: gesellschaftliche Missstände führen nicht notwendigerweise zu Protest der Betroffenen (Olsen 1965).

Wann formieren sich also soziale Bewegungen? Der Ressourcen-Mobilisierungsansatz (McCarthy und Zald 1977) betont die Notwendigkeit von Ressourcen wie Geld, Infrastruktur und Arbeit für das „Wachstum“ sozialer Bewegungsorganisationen – und die Rolle von BewegungsunternehmerInnen, die diese Ressourcen auftreiben. Wurde in der Mitte des 20. Jahrhunderts in den damals deutlich konservativeren Sozialwissenschaften Protest oftmals als abweichende Handlung sozialer AußenseiterInnen betrachtet, analysiert der Ressourcen-Mobilisierungsansatz Protestakteure als „rational“.

**Abb. 1:**
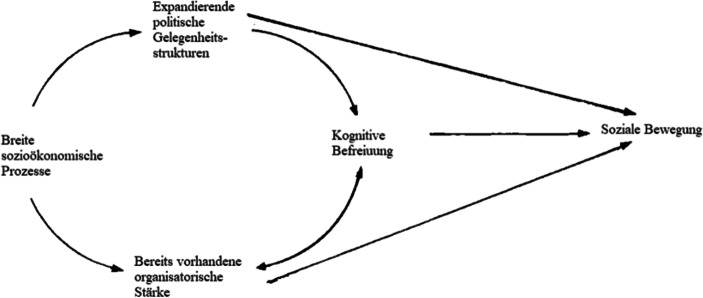
Das Politische-Prozess-Modell von McAdam (1982, S. 51)

Das „Politische-Prozess-Modell“ (McAdam 1982) baut u. a. auf diese Vorarbeiten auf und ist bis heute der bekannteste theoretische Erklärungsansatz zur Entstehung sozialer Bewegungen (siehe Abb. 1). Er betont strukturelle Faktoren. Vor dem Hintergrund breiter sozioökonomische Prozesse^3^ führt die Kombination aus expandierenden politischen Gelegenheitsstrukturen, bereits vorhandener organisatorischer Stärke und kognitiver Befreiung zur Entstehung sozialer Bewegungen.

„Politische Gelegenheitsstrukturen“ wurden in weiterer Folge als Erklärungsfaktor für die Entstehung (sowie auch für die Entwicklung und Erfolgsbedingungen) von sozialen Bewegungen umfangreich analysiert und konzeptualisiert. Bekannt ist ihre Operationalisierung anhand von vier Dimensionen: (1) „Die relative Offen- oder Geschlossenheit des institutionalisierten politischen Systems, (2) „die Stabilität von Elitekonstellationen, die typischerweise ein politisches System unterstützen“, (3) „die Anwesenheit von Verbündeten unter den politischen Eliten“ und (4) „die Möglichkeiten und der Willen des Staates zur Repression“ (McAdam et al. 1996). Konzeptuelle Weiterentwicklungen betrafen u. a. die Unterscheidung zwischen objektiven Gelegenheitsstrukturen und deren subjektiver Wahrnehmung (Kurzman 1996), die Relevanz anderer kontextueller Faktoren wie „diskursiven Gelegenheitsstrukturen“ (Koopmans und Statham 1999b) oder die Spezifizierung von relevanten Gelegenheitsstrukturen für unterschiedliche Bewegungen (Meyer und Minkoff 2004).

Auch jenseits der relativ unklar bleibenden „kognitiven Liberation“ im Politischen-Prozess-Modell hat sich die Forschung kulturellen Erklärungsansätzen zugewandt. Der Framing-Ansatz betont die Notwendigkeit von Kommunikation für Mobilisierung, beispielsweise beim Verknüpfen neuer Anliegen mit bekannten Sinnzusammenhängen (Snow et al. 1986) oder durch Handlungsaufrufe, welche das Erreichen eines Ziels als realistisch darstellen (Snow und Benford 1988). Ebenso ermächtigen kollektive Identitäten zum Aktivismus – deren Konstruktion bedarf jedoch langfristiger Arbeit, z. B. durch Abgrenzung und Erhöhung (Taylor und Whittier 1992).^4^

Ein gegen einen zu engen Fokus auf Strukturen formulierter Ansatz betont die *Agency* und strategische Entscheidungen von Protestakteuren als für ihre Entwicklung maßgeblich (Jasper 2004; Goodwin and Jasper 2011). Jenseits von bereits vorhandener organisatorischer Stärke wie dichten Netzwerken können sich AktivistInnen auch selbst rekrutieren, beispielsweise nach der emotionalen Erfahrung eines „moralischen Schocks“, der sie zum politischen Handeln drängt (Jasper und Poulsen 1995). Akteurszentrierte Ansätze betonen die Relevanz der Interaktion von AktivistInnen nicht mit „Strukturen“, sondern mit anderen Akteuren in bestimmten Arenen wie der Straße, Medien oder Gerichten, die von unterschiedlichen formellen und informellen Regeln geprägt sind (Jasper und Duyvendak 2015).

Auch die Befürworter struktureller Analysen begannen die Bedeutung von Interaktionen mit anderen Akteuren und wiederkehrende Mechanismen wie *brokerage* (das Vernetzen unterschiedlicher Akteure) oder *category formation *(das Bilden von Identitäten) als maßgeblich zu betrachten – und entwickeln in ihrem einflussreichen „contentious politics“-Ansatz eine relationale Perspektive (McAdam et al. 2001). Um der Entstehung und Entwicklung sozialer Bewegungen gerecht zu werden, bedarf es also multikausaler Erklärungsansätze. Opp (2009) versucht beispielsweise viele der oben genannten Perspektiven im Rahmen eines „strukturell-kognitiven Modells“ zu integrieren.

## Wie agieren soziale Bewegungen?

Die Beobachtung ihrer Aktionsformen ist notwendig, um eine soziale Bewegung zu identifizieren und zu analysieren. Der Kreativität sozialer BewegungsaktivistInnen sind kaum Grenzen gesetzt. Zu ihren Aktionsformen zählen z. B. Besetzungen, Brandstiftungen, Boykotts, Camps, Demonstrationen, Diskussionsveranstaltungen, das Hacken von Websites, Jagdstörungen, Lohnverhandlungen, Mahnwachen, Petitionen, das Schreiben von Broschüren oder politischen Programmen sowie Streiks. In Westeuropa entstand im Laufe des 18. und 19. Jahrhunderts ein modernes „Repertoire“ (Tilly 1978) an Protestformen (allen voran die Demonstration), welches sich laufend weiterentwickelte. Die Gegenüberstellung von „konventioneller“ Teilnahme an Wahlprozessen und sonstiger „unkonventioneller“ politischen Partizipation ist weiterhin geläufig – obwohl Straßenprotest lange nicht mehr als so unkonventionell gilt wie früher. Ebenso wird in der Literatur häufig zwischen „moderaten“ und „disruptiven“ Protestformen differenziert – letztere beeinträchtigen das Funktionieren institutioneller Abläufe (Piven 2008). Eine solche Differenzierung ist jedoch sehr kontextabhängig. Einfacher, aber grober ist die Unterscheidung zwischen „friedlichem“ und „gewalttätigem“ Protest.

Für die empirische Messung von Protestverhalten hat sich die Protestereignisanalyse (PEA) durchgesetzt. Sie liefert im Regelfall large-N-Daten auf Basis von Berichterstattung zu Protest in Tageszeitungen (Hutter 2014a). Für Westeuropa untersuchte die umfangreiche Forschung auf Basis von PEA-Daten die Relevanz von „Gelegenheitsstrukturen“ und zeigte beispielsweise, dass in Staaten, in denen ökonomische Konfliktlinien in der zweiten Hälfte des 20. Jahrhunderts besonders stark ausgeprägt waren, „neue soziale Bewegungen“ in deutlich geringerem Ausmaß mobilisieren konnten (Kriesi et al. 1995); dass der elektorale Erfolg rechtspopulistischer Parteien in den vergangenen Jahrzehnten mit besonders schwach ausgeprägten Rechtsaußen-Straßenprotest korrelierte (Giugni et al. 2005; Hutter 2014b); und dass im Kontext der Großen Rezession starke Straßenmobilisierung zu sozioökonomischen Themen negative Konsequenzen für die Wahlergebnisse etablierter Parteien hatte (Bremer et al. 2020).

Die PEA hat jedoch auch Limitierungen, etwa misst sie nicht notwendigerweise das tatsächliche Protestgeschehen: Medien berichten eher über Veranstaltungen mit vielen Teilnehmenden und über gewalttätigen Protest (Hutter 2014a). Ebenso fokussieren sie stark auf Ereignisse in Groß- und Hauptstädten. Um dieses Problem zu lindern, können Agenturmeldungen eine wichtige Quelle sein (Dolezal 2021). Gerade in einem föderalen Land wie Deutschland, zudem mit einem dezentralen Mediensystem, bleibt das Sammeln valider Daten eine Herausforderung (Wiedemann et al. 2022). Auch das politische Verhalten jenseits von Straßenprotesten bleibt unterbeleuchtet. Dagegen beziehen methodologische Innovationen wie die „political claims analysis“ (Koopmans und Statham 1999a) bzw. „contentious episode analysis“ (Bojar et al. 2021) den politischen Diskurs ein und bündeln Interaktionen.

Zudem kann die PEA bestimmte Aktionsformen, die entscheidend für das Verständnis vieler sozialer Bewegungen sind, nicht sinnvoll erfassen, da AktivistInnen eben nicht nur „auf der Straße“ agieren. Erstens betrifft das Online-Aktivismus (Castells 2015).^5^ Die „digitale Zivilgesellschaft“ boomt (Fitzpatrick 2018) und führt vor allem zu organisatorischen Erleichterungen (Earl und Kimport 2011; Kneuer und Richter 2015), wenn auch nicht zum Abbau interner Hierarchien (Gerbaudo 2012) oder der Vermeidung von Konflikt (Rone 2022a). Auch sind Rechtsaußen-Akteure in „sozialen“ Medien (Froio und Ganesh 2019; Gagnon 2020; Leidig 2021) und Online-Communities (Jasser et al. 2021) sehr aktiv oder zeigen sich als Produzenten „alternativer Medien“ (Rone 2022b). Zweitens sind auch die Veränderungen persönlicher Lebensstile (Haenfler et al. 2012) wie durch Veganismus (Cherry 2006) oder andere Formen politischen Konsums (Balsiger 2010) wichtige „private“ Aktionsformen. Drittens ist auch die Produktion medialer Inhalte „von unten“ eine Aktionsform, aber kein „Protestereignis“. Graswurzel-Medien sind auch auf gedrucktem Papier weiterhin von Relevanz (Weisskircher 2021), auch für die Rechtsaußen-Szene (Forchtner und Olsen 2023; Schilk 2017). Viertens kann auch die interne Organisation sozialer Bewegungsorganisation als Aktionsform verstanden werden, vor allem im Hinblick auf Innovationen im Bereich der Graswurzel-Demokratie (della Porta 2009; Doerr 2018). Die systematische Messung dieser Formen von Aktivismus kann durch Umfragedaten, auf Ebene von Individuen oder Organisationen, erfolgen.

Warum entscheiden sich Bewegungsakteure für bestimmte Aktionsformen? „Gelegenheitsstrukturen“ können auch hier entscheidend sein: fehlende formale Einflussmöglichkeiten auf politische Institutionen wie in Frankreich können Protest radikalisieren, während in relativ offenen Systemen wie der Schweiz moderate Protestformen überwiegen (Kitschelt 1986). Ähnlich wie bei der Frage nach der Entstehung von sozialen Bewegungen wird auch bei der Entscheidung für bestimmte Aktionsformen die Relevanz von agency, Protestkultur und „taste in tactics“ betont (Jasper 1997). Dabei entwickeln sich Aktionsformen durch strategische Interaktion mit anderen AkteurInnen wie z. B. GegnerInnen weiter (Jasper und Duyvendak 2015). Taktische Innovationen entstehen vor allem, wenn AktivistInnen besonders marginalisierte Anliegen vertreten (Wang und Soule 2016). Politischer Gewalt gehen oftmals komplexe Radikalisierungsprozesse voraus (della Porta 1995).

Ebenso können sich Protestakteure selbst schwierigen Bedingungen anpassen, z. B. im Kontext der Restriktion von Straßenprotest im Zuge der COVID-19-Pandemie (Flesher Fominaya 2022; Kowalewski 2021; Volk 2021). Sowohl dieses Beispiel als auch die zunehmende Relevanz von Online-Aktivismus im Allgemeinen zeigen, dass sich die „Repertoires“ sozialer Bewegungen laufend weiterentwickeln und die Analyse ihrer Aktionsformen kontinuierlich weitergedacht werden muss.

## Welchen Einfluss haben soziale Bewegungen?

Der Einfluss der vielfältigen Aktionsformen sozialer Bewegungen gehört wohl zu den empirisch relevantesten Untersuchungsobjekten in der Bewegungs- und Protestforschung – vor allem für die AktivistInnen selbst, geht es den Involvierten doch maßgeblich um das Durchsetzen politischer Anliegen. Dennoch sind die Beiträge in diesem Forschungsbereich noch immer vergleichsweise überschaubar, da Einfluss schwieriger zu messen ist als zum Beispiel Framing oder Protestereignisse. Entsprechende Studien haben sich daher lange mit der Konzeptualisierung von intendierten und nicht-intendierten Auswirkungen sowie Erfolg und Misserfolg beschäftigt. Dabei betonten sie, dass die hinter dem Einfluss liegenden Kausalketten höchstkomplex sind, Auswirkungen von vermeintlich ein und derselben „Bewegung“ in verschiedenen Politikfeldern und räumlichen Kontexten stark variieren und AktivistInnen oft geteilter Meinung sind, was die Interpretation von „Erfolg“ anbelangt – weshalb dieser erst recht schwierig zu analysieren ist (Giugni et al. 1999; Bosi et al. 2016).

In einer Pionierstudie unterscheidet Gamson (1975) zwischen zwei Arten von Auswirkungen: Aktivismus kann zur „Akzeptanz“ der HerausfordererInnen von Seiten politischer Eliten oder zu „neuen Vorteilen“ für die von den AktivistInnen vertretenen Gruppe führen. Seitdem betonte die Forschung eine Vielzahl an Effekten, auch jenseits der Gesetzgebung, z. B. Änderungen der öffentlichen Meinung (Banaszak und Ondercin 2016) und breiter kultureller Wandel (Amenta und Polelletta 2019), die Etablierung neuer Parteien (Kitschelt 1989), Einfluss auf Unternehmenspolitik und sogar auf Profite und Aktienkurse (Soule 2018), die Entwicklung neuer Technologien (Weisskircher 2019) oder gar Revolutionen und Regimewechsel (Goodwin 2001). Darüber hinaus hat Aktivismus auch Auswirkungen auf die AktivistInnen selbst, d. h. auf ihre politischen – und privaten – Biografien (McAdam 1988).

Wann ist Protest „erfolgreich“? Gamson (1975) sieht in seiner Analyse von „challenger groups“ in den Vereinigten Staaten von 1800 bis 1945 disruptive Taktiken, Organisation und die erfolgreiche Zentralisierung von Macht als entscheidend an. Auch Piven und Cloward (1977) argumentieren, dass disruptiver Protest für marginalisierte Gruppen erfolgsversprechend ist – doch bei einer Stärkung des Organisationsgrads und damit einhergehender Bürokratisierung würden sich AktivistInnen weniger disruptiven Aktionsformen bedienen. Goldstone (1980) kann in einer erneuten Analyse von Gamsons Datensatz jedoch die Signifikanz interner, organisatorischer Faktoren nicht bestätigen. Stattdessen verweist er auf nationale Krisen als entscheidenden strukturellen Grund für den Erfolg von AktivistInnen. Es ließe sich nun auf eine Vielzahl an Untersuchungen verweisen, die in bester sozialwissenschaftlicher Manier unterschiedliche Effekte für dieselben Variablen (z. B. gewalttätige Protestformen oder expandierende „Gelegenheitsstrukturen“) finden. Zauberformeln, die den Erfolg sozialer Bewegungen auf einen einfachen Nenner bringen können, sind von der Forschung kaum zu erwarten.^6^ Dafür verfolgen AktivistInnen zu unterschiedliche Ziele und wirken in zu unterschiedlichen, höchst komplexen historischen Kontexten. Jüngere Versuche möglichst generalisierender theoretischer Aussagen beschränken sich im Wesentlichen auf die nur bedingt überraschende These, dass Protest dann erfolgreicher ist, wenn Akteure ihre Strategien den kontextuellen Faktoren effektiv anpassen (Amenta 2006; McCammon 2012). Bereits Tilly (1978) merkt in Bezug auf die Erfolgsbedingungen von revolutionärer Mobilisierung an, bloß „oberflächliche Generalisierungen“ bieten zu können.

Ein Teil der Literatur wendet sich deshalb vom Fokus auf Korrelationen ab und betont die Rolle von Mechanismen und Interaktionen (Bosi und Uba 2021). Kolb (2007) unterscheidet zwischen verschiedenen Einflussmechanismen: Disruption, öffentliche Meinung, politischer Zugang, juristische Verfahren und internationale Politik. Je nach Kontext können diese in verschiedenen Kombinationen unterschiedliche Wirkung entfalten. Jasper et al. (2022) schlagen vor, jenseits großer und vermeintlich endgültiger Erfolge von Bewegungsakteuren die Vielzahl an *gains and losses*, die sie regelmäßig erfahren, zu erforschen. Der Alltag von AktivistInnen ist vom Bohren harter Bretter, keineswegs von Durchbrüchen, geprägt. Sie bewegen sich in verschiedenen Arenen, in Interaktion mit einer Vielzahl an PartnerInnen und GegnerInnen – und oftmals ist die Grenze zwischen diesen fließend. Dabei sind sie mit regelmäßig wiederkehrenden strategischen Dilemmata konfrontiert, wie: Ist für effektives politisches Handeln eine hierarchische Organisation notwendig, auch auf Kosten der Graswurzel-Demokratie? Sollen AktivistInnen disruptivere Aktionsformen verfolgen, um ihre Erfolgschancen zu vermehren, obwohl sie dadurch an öffentlicher Zustimmung verlieren oder gar staatlicher Repression ausgesetzt werden könnten? Sollen sie Allianzen mit mächtigeren Akteuren eingehen, in der Gefahr, von diesen vereinnahmt zu werden? Eine Untersuchung der mit diesen strategischen Dilemmata einhergehenden Vor- und Nachteile könnte die Forschung nach dem Einfluss sozialer Bewegungen auch für die AkteurInnen selbst relevanter machen.

## Forschungsperspektiven zu sozialen Bewegungen, Protest und Zivilgesellschaft

Die Bewegungs- und Protestforschung wird auch über die in diesem Artikel hinausgehenden Fragen immer vielfältiger, auch dank WissenschaftlerInnen aus dem deutschsprachigen Raum. Zur Vielzahl an weiteren spezialisierten Forschungsfeldern und -fragen gehören z. B. Transnationalisierungsprozesse (Doerr 2017; Knüpfer et al. 2022; Volk 2017), die Schwierigkeit von Allianzbildungen (Steinhilper 2021; Zajak und Haunss 2022), die Auswirkungen von Gegenprotest (Hager et al. 2021, Vüllers und Hellmeier 2022) und Repression (Grimm 2022; Opp und Roehl 1990; Reinisch 2023; Ullrich 2014), das Aufkommen von Rechtsaußen-Bewegungsparteien (Minkenberg 2019) sowie die Rolle von politischem Aktivismus in der internationalen Politik (Anderl et al. 2021; Holzscheiter 2020). Frühere deutschsprachige Beiträge zum Stand der Bewegungs- und Protestforschung boten explizite Ratschläge an die Forschungsgemeinschaft (Koopmans 1995; Rucht 2011; Teune 2008). Manchen davon wird Folge geleistet, zum Beispiel durch die Internationalisierung von theoretischen Debatten oder die Erforschung von Rechtsaußen-Bewegungen. Auch methodologisch erfolgen wichtige Beiträge, beispielsweise durch Demonstrationsbefragungen (Daphi et al. 2021), Ethnografie (Volk 2022) und Experimente (Hager et al. 2022).

Künftige Forschung sollte versuchen, die Bewegungs- und Protestforschung in einen größeren politikwissenschaftlichen und soziologischen Rahmen zu betrachten. McAdam und Boudet (2012) kritisieren Ansätze, die von einem „ptolemäischen Weltbild“ ausgehen, mit sozialen Bewegungen im Zentrum des Universums. Stattdessen plädieren sie für einen „kopernikanischen“ Zugang, der neben Protestierenden viele andere Faktoren, die Einfluss auf gesellschaftlichen Wandel ausüben, berücksichtigen (siehe auch Burstein 2014). Die enge Verknüpfung von Protestarena und Wahlarena (McAdam und Tarrow 2010; Kriesi et al. 2012) und die allgegenwärtige Präsenz von Protest in zeitgenössischen Gesellschaften (Jasper 2014) verlangen von Bewegungs- und ProtestforscherInnen, ihre Arbeit verstärkt mit der Analyse von Parteipolitik und gesellschaftlichen Entwicklungen zu verknüpfen. Ebenso würden Parteienforschung und politische Soziologie davon profitieren, in ihren Analysen sozialen Bewegungen verstärkt Platz einzuräumen.

Beispielsweise ist das politische System Deutschlands zunehmend von Politik auf der Straße geprägt: Manche in diesem Artikel genannten soziale Bewegungen brachten neue Themen in die politische Debatte ein, legten gar Grundsteine für die Entwicklung von Die Linke und der Alternative für Deutschland (AfD), und trugen zur wachsenden Polarisierung der Politik bei (Hutter und Weisskircher 2022). Ohne die Berücksichtigung der Kooperation von Teilen der AfD mit Protestgruppen – von PEGIDA über WindkraftgegnerInnen bis hin zu „Querdenken“ (Heinze und Weisskircher 2021, 2022) – ist die Entwicklung und Radikalisierung der Partei nicht zu verstehen. Fridays for Future ist ein Beispiel für Protest, der intensive und widersprüchliche Parteireaktionen hervorruft und die öffentliche Debatte mitbestimmt (Berker und Pollex 2021; Heinze 2023). Diese Fälle zeigen bereits, dass Parteienforschung und Protestforschung kaum voneinander getrennt gedacht werden können. Für umfassende Perspektiven sollte künftige Forschung die enge Beziehung von Protest- und Wahlarena berücksichtigen.

Ebenso gilt es, die Bewegungs- und Protestforschung mit Gesellschaftstheorie und -analyse zu verknüpfen.^7^ Die wachsende Relevanz sozialer Bewegungen reflektiert defizitäre Entwicklungen in unseren politischen Systemen und Gesellschaften.^8^ Wie lässt sich Politik auf der Straße mit Diagnosen des Endes von Volksparteien (Koß 2021) oder gar von „Postdemokratie“ (Crouch 2004) verknüpfen? Spiegeln die Komposition und die Forderungen zeitgenössischer sozialer Bewegungen eine „Abstiegsgesellschaft“ (Nachtwey 2016), eine „Gesellschaft der Singularitäten“ (Reckwitz 2017) oder das „Ende der Illusionen“ (Reckwitz 2019) wider? Inwiefern reflektiert und reproduziert die Protestarena objektive und subjektive Unterschiede zwischen Stadt und Land (Haffert 2022) und West und Ost (Mau 2019, Pickel und Pickel 2022)? Wie tragen die Online-Aktivitäten sozialer Bewegungen zu „einem erneuten Strukturwandel der politischen Öffentlichkeit“ (Habermas 2021) bei? Die Analyse sowohl von (Partei‑)Politik als auch von gesellschaftlichen Makro-Entwicklungen würde von einer verstärkten Berücksichtigung sozialer Bewegungen, einem der wichtigsten Motoren des sozialen Wandels, profitieren.

## Footnotes

1 Auf Grund dieser Herausforderung betont Burstein (1999) wohl folgerichtig, dass konzeptuell keine klare Trennlinie zwischen sozialen Bewegungen, Interessengruppen und politischen Parteien gezogen werden kann. Was Organisations- oder Aktionsformen anbelangt sind die Übergänge zwischen diesen Akteurstypen fließend: manche Parteien sind nicht nur loser organisiert als bestimmte soziale Bewegungsorganisationen, sondern nehmen sogar häufiger an Straßenprotesten teil. Selbst politische Parteien zeichnen sich nicht durch eine eindeutige sozialwissenschaftliche Definition aus, sondern eher dadurch, dass sie in vielen Staaten als eigene rechtliche Kategorie gelten.

2 Bereits lange vor der Institutionalisierung der Bewegungs- und Protestforschung gab es reges wissenschaftliches Interesse an sozialen Bewegungen (z. B. Marx 1852/2007; Hobsbawm 1959; Tocqueville 1835/1840/1986). Für die Betonung „europäischer“ Perspektiven vor der Kanonisierung des US-dominierten empirisch-analytischen Ansatzes siehe Cox und Flesher Fominaya (2013).

3 Nicht nur bei McAdam sind diese „breiten sozioökonomischen Prozesse“ nicht mehr primäres Forschungsinteresse – manche WissenschaftlerInnen beklagen deshalb das „eigenartige Verschwinden des Kapitalismus in der Bewegungsforschung“ (Hetland und Goodwin 2013).

4 Dagegen bleiben „Klassen ohne Bewusstsein“ demobilisiert (Westheuser und della Porta 2022).

5 In einem Zeitalter, in dem die Online-Sphäre von zunehmender Bedeutung ist, gilt es die schon oftmals vergessene, aber oben zitierte, Konzeptualisierung von sozialen Bewegungen als “a set of opinions and beliefs in a population” (McCarthy und Zald 1977, S. 1217–1218) in Erinnerung zu rufen.

6 Die medientauglichen 3,5 % der Bevölkerung, die genügen würden, durch gewaltfreien Protest DiktatorInnen zu Fall zu bringen (Chenoweth 2013; Chenoweth und Belgioioso 2019), wurden von Extinction Rebellion zum Zwecke ihres Aktivismus in völlig anderen politischen Kontexten übernommen und somit missverstanden (Matthews 2020).

7 Diesen Punkt betonte auch Dieter Rucht im Rahmen der Tagung „Spaziergang zum Umsturz: Sozialwissenschaftliche Perspektiven auf die Querdenken-Proteste“ (Hamburger Institut für Sozialforschung, Juni 2022).

8 Eine solche Perspektive ist erst recht von Bedeutung, wenn Forschung zu Protest zunehmend von staatlicher oder privatwirtschaftlicher Förderung abhängig ist, die möglicherweise nicht primär von einem selbstkritischen Impetus geprägt ist (Teune und Ullrich 2018).
